# The Association between Serum Creatinine/Cystatin C Ratio and Cardiovascular Morbidity and Mortality: Insights from NHANES

**DOI:** 10.31083/j.rcm2409275

**Published:** 2023-09-25

**Authors:** Jianli Shi, Yufeng Wu, Shiyu Zhu, Yao Xie, Meixiang Xiang

**Affiliations:** ^1^Department of Cardiology, Second Affiliated Hospital, Zhejiang University School of Medicine, 310000 Hangzhou, Zhejiang, China

**Keywords:** serum creatinine/cystatin C ratio, muscle mass loss, cardiovascular diseases, cardiovascular morbidity, cardiovascular mortality, the National Health and Nutrition Examination Survey

## Abstract

**Background:**

The Serum creatinine/cystatin C ratio (Cr/CysC ratio) is an emerging alternative index for muscle mass loss, a risk factor for cardiovascular diseases (CVDs). However, the association between the Cr/CysC ratio and CVD morbidity and mortality remains unknown.

**Methods:**

A total of 11,150 participants of the National Health and Nutrition Examination Survey (NHANES) were included in this study. Univariable and multivariable logistic regression models were employed to assess the association between the Cr/CysC ratio and self-reported CVD morbidity. Cox proportional hazard models were used to estimate the hazard ratio (HR) and 95% confidence interval (CI) of the Cr/CysC ratio for CVD mortality.

**Results:**

At baseline, 1181 (7.90%) participants had self-reported CVDs. Lower Cr/CysC ratios were found in participants with CVDs (1.18 ± 0.30 vs. 1.05 ± 0.23, *p* < 0.001). In the multivariable logistic regression model, the Cr/CysC ratio was inversely linked to CVD morbidity (odds ratio: 0.65, 95% CI: 0.52–0.81, *p* < 0.001, per standard deviation [SD] increase). 997 (8.94%) CVD deaths were documented during a median follow-up of 16.9 years. A higher Cr/CysC ratio was associated with a decreasing risk of CVD mortality (adjusted HR: 0.54, 95% CI: 0.46–0.65, *p* < 0.001, per SD increase).

**Conclusions:**

In NHANES participants, the Cr/CysC ratio had an inverse correlation with CVD morbidity and mortality.

## 1. Introduction

Cardiovascular diseases (CVDs), which contribute about 40% to all-cause mortality, constitute a leading worldwide health problem [1]. Since the mid-20th century, cardiovascular risk factors have been widely studied, but the prediction and prevention of CVD are still challenging [2].

Muscle mass loss is prevalent in the elderly population [3]. CVD patients are more likely to suffer from low muscle mass [4]. Recent studies have illustrated the predictive value of muscle mass in all-cause mortality in CVD patients [5, 6, 7]. Therefore, screening and diagnosis of low muscle mass. Current assessment methods for assessing muscle mass, such as computed tomography scans [8] and bioelectrical impedance analysis [9], are inconvenient, costly, and time-consuming. As a result, applying these methods to examine muscle mass in critically ill patients and the elderly seems impractical. Therefore, there is a need to establish more convenient diagnostic criteria for defining low muscle mass.

The serum creatinine/cystatin C ratio (Cr/CysC ratio) is readily available and is an alternative index for determining muscle mass. Since creatinine is mainly released from muscle tissue, body muscle mass is positively correlated with serum creatinine concentrations [10]. Cystatin C, which is universally generated by nucleated cells, is not influenced by muscle mass [11]. Numerous studies have demonstrated significant correlations between the Cr/CysC ratios and muscle mass in diabetes, lung transplant candidates, and cancer [12, 13, 14, 15], as well as the prognostic value of the Cr/CysC ratio in patients with non-dialysis chronic kidney disease or chronic obstructive pulmonary disease [16, 17]. Nevertheless, the relationship between the Cr/CysC ratio and CVD morbidity and mortality remains unclear.

As a result, we sought to investigate whether the Cr/CysC ratio is related to CVD morbidity and mortality using the data from the National Health and Nutrition Examination Survey (NHANES).

## 2. Materials and Methods

### 2.1 Study Population

NHANES is a national survey which utilizes a complex, multistage probability sampling design to analyze the nutritional and health conditions of civilians in the United States every two years. Home-interviews were conducted and participants were invited to the mobile examination center (MEC) for various physiologic examinations and blood tests. The NHANES study was authorized by the National Center for Health Statistics Research Ethics Review Board (Hyattsville, MD, USA). Informed consent was signed by all participants. Detailed descriptions of NHANES and guidance on analytical approaches are available at https://www.cdc.gov/nchs/nhanes/index.htm.

**Fig. 1.**
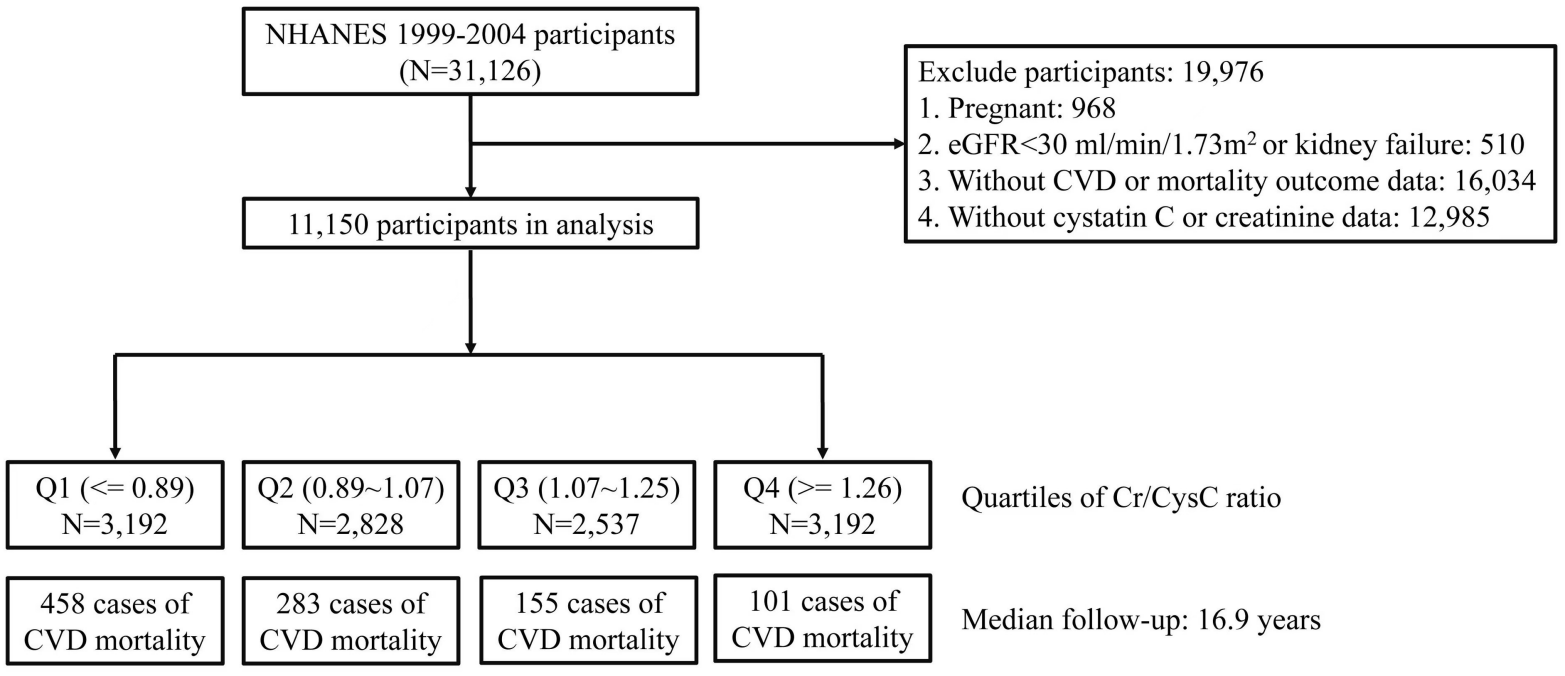
**Flow chart of the study**. NHANES, National Health and Nutrition Examination Survey; eGFR, estimated glomerular filtration rate; CVD, cardiovascular disease; N, number.

Data from NHANES during 1999–2004 (N = 31,126) were analyzed in this cohort study. We excluded pregnant participants (N = 968), those without CVD morbidity or mortality outcome information (N = 16,034), or those without cystatin C or creatinine data (N = 12,985). Since serum creatinine and cystatin C levels are influenced by renal function [18], we also excluded participants with estimated glomerular filtration rate (eGFR) <30 mL/min/1.73 $m^{2}$ or kidney failure (N = 510). Overall, 19,976 participants were excluded from our study. The analysis consisted of 11,150 participants, as shown in Fig. 1.

### 2.2 Measurement of Serum Creatinine and Cystatin C

Blood samples were taken, centrifuged, and then stored at <–20 °C prior to analysis. Serum creatinine was measured using the kinetic Jaffe method. Regression models have been developed based on the National Center for Health Statistics to adjust for serum creatinine values in NHANES 1999–2000 [19]. Surveys conducted in 2001–2002 and 2003–2004 did not need any corrections. A Cystatin C immunoassay (Siemens Healthcare Diagnostics) was used to measure Cystatin C in serum.

### 2.3 Determination of Cardiovascular Morbidity and Mortality

CVDs were determined by self-reported physician diagnosis through a home interview using a standardized questionnaire. “Has a doctor or other health expert ever told you that you have heart failure (HF)/coronary heart disease (CHD)/angina/myocardial infarction (MI)/a stroke?” the researchers asked the participants. Participants were considered to have CVD if they answered “yes” to one of the questions. A probabilistic match was used to derive death outcomes for each participant by linking to the National Death Index (NDI) until December 31, 2019. The cause of death was determined using code of the International Classification of Diseases (ICD-10). Cardiovascular mortality included death due to cardiac disease (I00–I09, I11, I13, and I20–I51) and cerebrovascular disease (I60–I69). The follow-up time was counted from the date of home interview to the date of death or December 31, 2019, whichever came first. Further details can be found at https://www.cdc.gov/nchs/data-linkage/mortality-public.htm.

### 2.4 Assessment of Covariates

Demographic data (age, gender, and ethnicity) were acquired during home interviews. Alcohol consumption and smoking were obtained via self-reporting. Diagnosis of hypertension included self-reported medical diagnosis, the use of blood pressure medications, or measured blood pressure of ≥140/90 mmHg. The definition of diabetes included self-reported medical diagnosis, any application of antidiabetic treatment, or a glycated hemoglobin A1c (HbA1c) level of more than 6.5%. Hyperlipidemia was defined as a history of hypercholesterolemia, the use of lipid-lowering agents or or low-density lipoprotein cholesterol (LDL-C) ≥4.14 mmol/L. Body mass index (BMI) was calculated as the weight in kilograms divided by the square of the height in metres. Appendicular lean mass (ALM) was refered to the combined fat-free mass of all limbers. ALM and ALM divided by BMI were representative of muscle mass on the basis of the Foundation for the National Institutes of Health [20].

Laboratory results, including baseline biochemical tests, lipid profiles, and HbA1c, were measured during the MEC examination in line with standard protocols. The LDL-C was calculated using the Friedewald equation [21]. The equation of Chronic Kidney Disease Epidemiology Collaboration (CKD-EPI) was applied to calculate the estimated glomerular filtration rate (eGFR) [22].

### 2.5 Statistical Analysis

Given the complex survey design, proper sample weights were employed during all analyses according to NHANES analytic guidelines. Continuous variables with normal distribution were expressed as mean ± standard deviation (SD) while those with a skewed distribution were shown as median (interquartile range, IQR). Categorical variables were manifested as numbers (percentage, %). Chi-square test was utilized to analyze categorical variables as well as Kruskal-Wallis test for continuous variables. In addition, we performed univariable linear regression analysis to display the relationship between the Cr/CysC ratio and ALM or ALM divided by BMI.

The relationship between the Cr/CysC ratios and total CVD or individual CVD types was investigated via univariable and multivariable logistic regression models. We adjusted the multivariable model for age, gender, ethnicity, eGFR, as well as traditional cardiovascular risk factors (smoking, BMI, systolic blood pressure (SBP), LDL-C, and HbA1c).

Cumulative incidence was estimated with Kaplan–Meier analyses and log-rank tests. The Cox proportional hazard model for CVD mortality was adjusted for sex, age, ethnicity, SBP, LDL-C, BMI, HbA1c, smoking, eGFR, as well as HF, angina pectoris, MI, CHD, and stroke at baseline. The Cr/CysC ratio was modeled as a continuous variable or categorical groups.

We performed subgroup analysis according to age, gender, race, smoking, CVD, BMI, hypertension, diabetes, eGFR, and hyperlipidemia. We used the same variables for adjustment as in the Cox proportional hazard models. Potential interactions were also tested.

The “survey” package (version 4.1-1) for R statistical software (version 4.1.1, R Foundation for Statistical Computing, Vienna, Austria) was applied for all statistical analysis. All statistical tests were two-tailed. A *p*-value less than 0.05 was regarded as statistically significant.

## 3. Results

### 3.1 Population Characteristics

In Table 1, we present the characteristics of the participants. Among all participants, 49% were men, and the average age was 44. The CVD morbidity in participants with higher Cr/CysC ratios was lower than that in participants with lower Cr/CysC ratios [3.3% in quartile 4 (Q4, ≥1.26) vs. 14% in quartile 1 (Q1, ≤0.89)]. Significant differences in uric acid, cholesterol, BMI, HbA1c, ethnicity, diabetes, hypertension, hyperlipidemia, BMI, and smoking were found among participants with different quartiles of Cr/CysC ratios (all *p* < 0.05).

**Table 1.** **Population characteristics stratified by Cr/CysC ratio**.

Characteristics	Overall	Q1 (≤0.89)	Q2 (0.89~1.07)	Q3 (1.07~1.25)	Q4 (≥1.26)	*p*-value
	N = 11,150	N = 3192	N = 2828	N = 2537	N = 2593	
Sociodemographic						
Age, year	44 (33, 57)	54 (40, 69)	46 (34, 60)	41 (31, 52)	39 (30, 48)	<0.001
Gender						<0.001
Male	5664 (49%)	717 (19%)	1271 (40%)	1598 (58%)	2078 (79%)	
Female	5486 (51%)	2475 (81%)	1557 (60%)	939 (42%)	515 (21%)	
Ethnicity						<0.001
Non-Hispanic White	5773 (72%)	1803 (77%)	1546 (75%)	1336 (72%)	1088 (65%)	
Mexican American	2481 (7.2%)	858 (7.4%)	668 (7.3%)	527 (7.1%)	428 (7.0%)	
Other Hispanic	513 (5.7%)	164 (6.6%)	122 (5.4%)	110 (5.6%)	117 (5.2%)	
Non-Hispanic Black	2011 (11%)	275 (5.5%)	395 (8.1%)	473 (10%)	868 (19%)	
Others	372 (4.4%)	92 (4.1%)	97 (4.5%)	91 (4.5%)	92 (4.4%)	
Self-reported medical conditions						
Heart failure	301 (1.9%)	146 (4.0%)	80 (2.0%)	49 (1.1%)	26 (0.6%)	<0.001
Coronary heart disease	467 (3.2%)	184 (4.9%)	119 (3.3%)	106 (2.9%)	58 (1.7%)	<0.001
Angina pectoris	389 (2.8%)	163 (4.5%)	114 (3.3%)	75 (2.2%)	37 (1.1%)	<0.001
Myocardial infarction	478 (3.2%)	197 (5.1%)	119 (3.2%)	102 (3.0%)	60 (1.5%)	<0.001
Stroke	355 (2.3%)	163 (4.4%)	92 (2.2%)	69 (1.7%)	31 (0.7%)	<0.001
Cardiovascular disease	1182 (7.9%)	509 (14%)	311 (8.3%)	237 (6.4%)	125 (3.3%)	<0.001
Diabetes	1401 (8.8%)	553 (13%)	376 (9.6%)	269 (6.7%)	203 (5.7%)	<0.001
Hypertension	4615 (35%)	1723 (48%)	1220 (36%)	897 (29%)	775 (25%)	<0.001
Hyperlipidemia	3842 (33%)	1250 (39%)	1026 (35%)	794 (29%)	772 (30%)	0.002
Smoking	5519 (50%)	1586 (54%)	1482 (52%)	1280 (48%)	1171 (44%)	<0.001
Drinking	4808 (58%)	902 (42%)	1170 (55%)	1279 (65%)	1457 (70%)	<0.001
Examination						
SBP, mmHg	123 ± 19	129 ± 22	124 ± 20	121 ± 17	121 ± 15	<0.001
DBP, mmHg	72 ± 13	70 ± 14	72 ± 12	73 ± 12	73 ± 12	<0.001
BMI, kg/$m^{2}$	28.1 ± 6.2	29.6 ± 7.5	28.3 ± 6.4	27.3 ± 5.6	27.1 ± 4.9	<0.001
Waist circumference, cm	96.2 ± 15.5	98.9 ± 16.7	96.8 ± 16.1	95.1 ± 15.0	94.1 ± 13.4	<0.001
Laboratory test						
Total cholesterol, mmol/L	5.18 ± 1.07	5.30 ± 1.09	5.19 ± 1.04	5.14 ± 1.09	5.11 ± 1.05	<0.001
Triglycerides, mmol/L	1.25 (0.85, 1.87)	1.41 (0.97, 2.03)	1.30 (0.86, 1.94)	1.22 (0.82, 1.85)	1.08 (0.74, 1.64)	<0.001
HDL-C, mmol/L	1.35 ± 0.40	1.36 ± 0.41	1.36 ± 0.41	1.33 ± 0.40	1.34 ± 0.39	0.045
LDL-C, mmol/L	3.10 ± 0.96	3.13 ± 1.01	3.09 ± 0.91	3.08 ± 0.96	3.11 ± 0.97	0.177
Uric acid, umol/L	319.5 ± 84.5	309.2 ± 85.6	308.8 ± 84.8	322.3 ± 84.9	337.8 ± 79.2	<0.001
HbA1c, %	5.46 ± 0.88	5.61 ± 1.00	5.49 ± 0.91	5.38 ± 0.76	5.37 ± 0.82	<0.001
eGFR, mL/min/1.73 $m^{2}$	95.0 ± 21.5	94.7 ± 24.0	97.2 ± 22.6	96.5 ± 20.4	91.6 ± 18.1	<0.001
Creatinine, mg/dL	0.89 ± 0.20	0.77 ± 0.19	0.84 ± 0.17	0.91 ± 0.17	1.02 ± 0.17	<0.001
Cystatin C, mg/dL	0.78 ± 0.19	0.90 ± 0.23	0.78 ± 0.16	0.74 ± 0.14	0.68 ± 0.11	<0.001
Cr/CysC ratio	1.17 ± 0.30	0.86 ± 0.10	1.07 ± 0.05	1.23 ± 0.05	1.51 ± 0.34	<0.001

Abbreviations: SBP, systolic blood pressure; DBP, diastolic blood pressure; BMI, body mass index; Cr, creatinine; CysC, cystatin C; LDL-C, low-density lipoprotein-cholesterol; HDL-C, high-density lipoprotein-cholesterol; HbA1c, glycated hemoglobin A1c; eGFR, estimated glomerular filtration rate; N, number.

In addition, the Cr/CysC ratio was positively related with muscle mass index, including ALM and ALM to BMI (Table 2, *p* < 0.001).

**Table 2.** **Univariate Linear Regression for muscle mass and Cr/CysC ratio**.

	Univariate Linear Regression	95% CI	*p*-value
	Beta		
ALM/BMI, kg/kg*$m^{–\,2}$	0.654	0.626, 0.682	<0.001
ALM, kg	0.015	0.013, 0.016	<0.001

Abbreviations: ALM, appendicular lean mass; BMI, body mass index; CI, confidence interval; Cr, creatinine; CysC, cystatin C.

### 3.2 Cr/CysC Ratio and CVD Morbidity

The association between the Cr/CysC ratio and the incidence of CVD was exhibited in Table 3. In the univariable model, the odds ratio (OR) of the Cr/CysC ratio for total CVDs was 0.50 (95% CI: 0.45–0.56, *p* < 0.001). After controlling for demographic variables, traditional cardiovascular risk factors and eGFR, the correlation remained significant (OR: 0.65, 95% CI: 0.52–0.81, *p* < 0.001). We further analyzed the relationship between the Cr/CysC ratio and CVD morbidity by gender, age, malignancy and inflammation, and obtained the similar results (**Supplementary Tables 1,2,3,4**). According to the univariable model, significant associations were found between the Cr/CysC ratios and individual types of CVDs, including HF, CHD, angina, MI, and stroke (all *p* < 0.01). However, after adjusting for all variables, a significant correlation was only found between the Cr/CysC ratio and HF, angina, MI, and stroke (all *p* < 0.05).

**Table 3.** **Univariate and multivariate logistic regression model of Cr/CysC ratio (per SD increase) for CVD morbidity**.

Outcomes	Unadjusted model		Adjusted ${\mathrm{model}}^{a}$	
	${\mathrm{OR}}^{b}$ (95% CI)	*p*-value	${\mathrm{OR}}^{b}$ (95% CI)	*p*-value
CVD	0.50 (0.45, 0.56)	<0.001	0.65 (0.52, 0.81)	<0.001
Heart failure	0.39 (0.33, 0.46)	<0.001	0.38 (0.28, 0.52)	<0.001
Coronary heart disease	0.62 (0.54, 0.73)	<0.001	0.88 (0.70, 1.12)	0.296
Angina pectoris	0.54 (0.46, 0.64)	<0.001	0.76 (0.58, 0.98)	0.035
Myocardial infarction	0.59 (0.50, 0.71)	<0.001	0.71 (0.51, 0.97)	0.035
Stroke	0.44 (0.35, 0.54)	<0.001	0.49 (0.35, 0.67)	<0.001

^a^Adjusted for age, gender, and ethnicity, SBP, LDL-C, BMI, HbA1c, smoking, eGFR. ^b^Odds ratio of Cr/CysC ratio (per SD increase). Due to missing data, 768 participants are excluded from the adjusted model. Abbreviations: OR, odds ratio; CI, confidence interval; Cr, creatinine; CysC, cystatin C; BMI, body mass index; SBP, systolic blood pressure; LDL-C, low-density lipoprotein-cholesterol; HbA1c, glycated hemoglobulin A1c; eGFR, estimated glomerular filtration rate; CVD, cardiovascular disease.

### 3.3 Cr/CysC Ratio and CVD Mortality

**Fig. 2.**
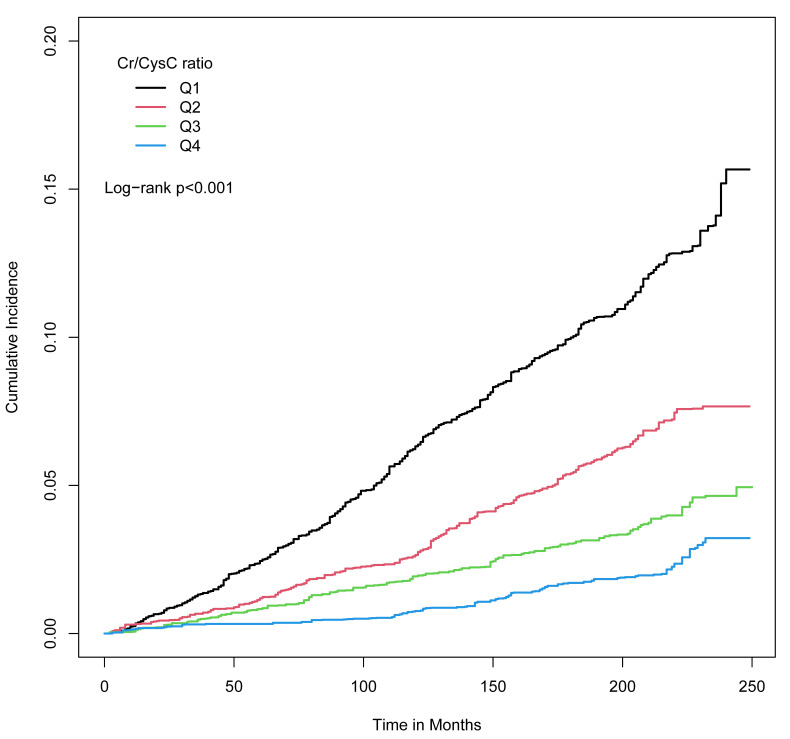
**Cumulative incidence of CVD mortality**. Kaplan-Meier curves were depicted according to Cr/CysC ratio quartiles. Log-rank *p* < 0.001. CVD, cardiovascular disease; Cr, creatinine; CysC, cystatin C.

During a median follow-up of 16.9 years (173,344 person-years), 997 records (8.94%) of CVD death were documented. As is shown in the Kaplan-Meier curves, participants with the Cr/CysC ratio ≥1.26 suffered from lower CVD mortality (Fig. 2). The trends were the similar for participants of different ages and genders (**Supplementary Fig. 1** and **Supplementary Fig. 2**). Compared with participants whose Cr/CysC ratio ≤0.89, the multivariable-adjusted hazard ratio (HR) (95% CI) for those whose Cr/CysC ratio ≥1.26 was 0.37 (95% CI: 0.26–0.54) for CVD mortality. A higher Cr/CysC ratio (per standard deviation) was associated with decreasing risk of CVD mortality (adjusted HR: 0.54, 95% CI: 0.46–0.65, *p* < 0.001, per SD increase) (Table 4). Whether suffering from inflammation or malignancy, the relationship between the Cr/CysC ratio and CVD mortality was significant (**Supplementary Table 5**).

**Table 4.** **Cox proportional hazards model for CVD mortality**.

Cr/CysC ratio range	Cr/CysC ratio quartiles				Continuous
	Q1	Q2	Q3	Q4	Per standard deviation greater
	(≤0.89)	(0.89~1.07)	(1.07~1.25)	(≥1.26)	
Events/N at risk	458/3192	283/2828	155/2537	101/2593	997/11,150
Unadjusted HR (95% CI)	1.00 (Ref.)	0.53 (0.44, 0.64)	0.30 (0.25, 0.37)	0.18 (0.14, 0.22)	0.41 (0.37, 0.45)
Adjusted Model* HR (95% CI)	1.00 (Ref.)	0.68 (0.54, 0.85)	0.53 (0.40, 0.70)	0.37 (0.26, 0.54)	0.54 (0.46, 0.65)

*Adjusted for sex, age, ethnicity, SBP, LDL-C, BMI, HbA1c, smoking, eGFR, having HF, angina pectoris, MI, CHD, and stroke. Due to missing data, 768 participants are excluded from the adjusted model. Events and numbers at risk were unweighted; HR, hazard ratio. CVD, cardiovascular disease; Cr, creatinine; CysC, cystatin C; HR, hazard ratio; HF, heart failure; MI, myocardial infarction; CHD, coronary heart disease; N, number; SBP, systolic blood pressure; LDL-C, low-density lipoprotein cholesterol; BMI, body mass index; HbA1c, glycated hemoglobin A1c; eGFR, estimated glomerular filtration rate.

### 3.4 Subgroup Analysis

**Fig. 3.**
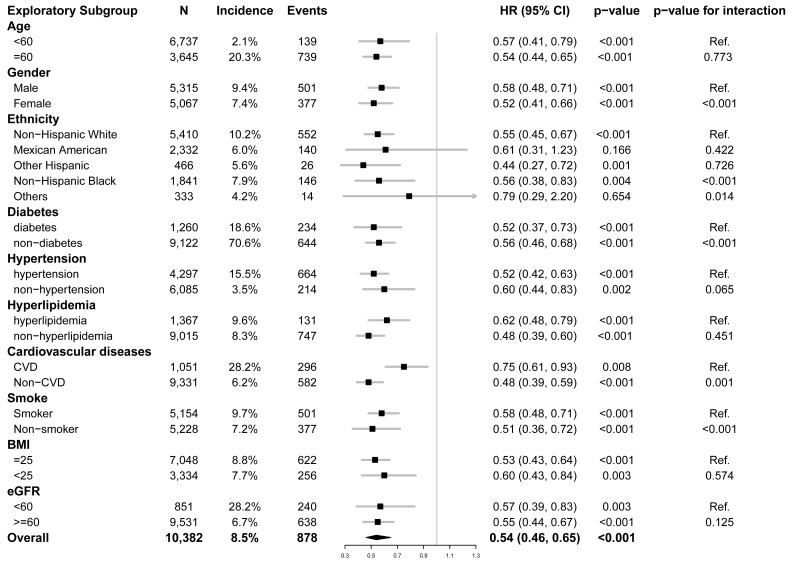
**Subgroup analysis of the association of Cr/CysC ratio (per 1 SD) with CVD mortality**. N, number; HR, hazard ratio; CVD, cardiovascular disease; BMI, body mass index; eGFR, estimated glomerular filtration rate; Cr, creatinine; CysC, cystatin C.

Fig. 3 shows a stratified analysis of the Cr/CysC ratio and CVD mortality risks. Results of stratified analyses were almost consistent with those from the overall analysis, except for Mexican Americans and other ethnicities. Significant interactions were found in diabetes, gender, smoking, and baseline CVD. The Cr/CysC ratio had a greater impact on cardiovascular mortality among females, diabetics, non-smokers, non-CVDs and non-Hispanic black participants.

## 4. Discussion

In this nationwide survey, we found: (1) There was an inverse relationship between the Cr/CysC ratio and the CVD morbidity. (2) A decreasing trend of risk for CVD mortality was found in participants with a higher Cr/CysC ratio.

The effects of low muscle mass are detrimental to life quality, financial burden, as well as health care costs [23]. As previous measurements of muscle mass were not readily available, a more convenient and feasible screening method was urgently required [24, 25]. Tetsuka first observed an association between muscle function and the Cr/CysC ratio in 2013. Individuals with amyotrophic lateral sclerosis had a lower Cr/CysC ratio than healthy controls [26]. A few previous studies have explored the association between muscle mass and CVD risk, and mortality [24, 27, 28]. The Cr/CysC ratio was proved to be relevant to major adverse cardiovascular events (MACE) in several studies. In 2021, Lu *et al*. [29] discovered that a lower Cr/CysC ratio was an independent risk factor for MACE among patients suffering from obstructive coronary artery disease. The lower Cr/CysC ratio in patients with type 2 diabetes mellitus predicted a lower brachial-ankle pulse wave velocity and a higher incidence of subclinical atherosclerosis [30]. Although muscle mass loss is associated with an increased incidence of CVD and mortality, it is unclear whether the Cr/CysC ratios are related to CVD mortality and morbidity. Our study demonstrated the link between the Cr/CysC ratio and cardiovascular prevalence and mortality for the first time.

The possible explanations for the relevance between the Cr/CysC ratio and CVD morbidity and mortality are discussed below. First, besides contractile function, skeletal muscle was also an important metabolic [31] and endocrine [32] organ, which secreted a wide range of active myokines to regulate energy consumption, insulin sensitivity and protein synthesis. Consequently, muscle mass loss may not only be a complication of aging, physical inactivity, or HF, but also cause some metabolic [33] and age-related myocardial diseases [34]. Second, low muscle mass often co-exists with cardiovascular risk factors. Previous studies have confirmed that muscle mass was independently related to hypertension [35], depression [36], and diabetes [37]. Physical inactivity and long bed rest may be the main factors for the reduction of muscle mass and strength. Third, in addition to body muscle mass, white blood cell count and protein intake were also associated with serum creatinine levels [38]. Furthermore, Cystatin C levels were higher in diabetics and those with chronic inflammation [39], so Cr/CysC ratio may represent the inflammatory or nutritional status.

## 5. Conclusions

Our study demonstrated that increased Cr/CysC ratios were associated with a lower CVD morbidity and mortality. Serum Cr/CysC ratio, a promising indicator of muscle mass, may have predictive value for the prognosis of CVDs and can be applied clinically to assess cardiovascular risk in the elderly and severely ill patients.

## 6. Strengths and Limitations

As far as we know, our study is the first prospective cohort to investigate the link between the Cr/CysC ratios and CVD morbidity and mortality. A major advantage of our study is the large sample size from the nationally-representative cohort of the US, which allowed us to perform the stratified analysis with sufficient statistical power. The stratified analysis showed that the impact of the Cr/CysC ratio on CVD mortality was stronger in the female, diabetic, non-smoker, and other Hispanic subgroups.

This study has some limitations. First, CVDs were self-reported rather than documented in medical records, which resulted in recall bias. Second, the link between the Cr/CysC ratio and CVD morbidity was assessed using cross-sectional data. This association requires further study by cohorts or experimental designs. Third, only mortality data was available in NAHNES, restricting us from investigating the longitudinal association between the Cr/CysC ratio and major CVDs such as HF, stroke, and MI. Finally, the results might also be influenced by residual confounders, random errors, or uncontrolled factors, even after adjusting for many possible confounders.

## Data Availability

Detailed descriptions of NHANES, all data, and guidance on analytical approaches can be found at https://www.cdc.gov/nchs/nhanes/index.htm.

## Abbreviations

Cr/CysC ratio, creatinine/cystatin C ratio; CVDs, cardiovascular diseases; CT, computed tomography; NHANES, the National Health and Nutrition Examination Survey; MEC, mobile examination center; SBP, systolic blood pressure; DBP, diastolic blood pressure; HbA1c, hemoglobin A1c; BMI, body mass index; ALM, appendicular lean mass; FNIH, the Foundation for the National Institutes of Health; LDL-C, low-density lipoprotein cholesterol; eGFR, glomerular filtration rate; SD, standard deviation; IQR, interquartile range; HF, heart failure; CHD, coronary heart disease; MI, myocardial infarction; OR, odds ratio; MACE, major adverse cardiovascular events; HR, hazard ratio; CI, confidence interval; SD, standard deviation.

## Supplementary Material

Supplementary material associated with this article can be found, in the online version, at https://doi.org/10.31083/j.rcm2409275.
