# Supplementary material for: The Association between Serum Creatinine/Cystatin C Ratio and Cardiovascular Morbidity and Mortality: Insights from NHANES
Source: Rev Cardiovasc Med. 2023 Sep 25;24(9):275. doi: 10.31083/j.rcm2409275 (PMC11270077; doi:10.31083/j.rcm2409275)
Supplement: Supplementary file 1 [file 2153-8174-24-9-275-s1.zip › 2153-8174-24-9-275-s1.docx]

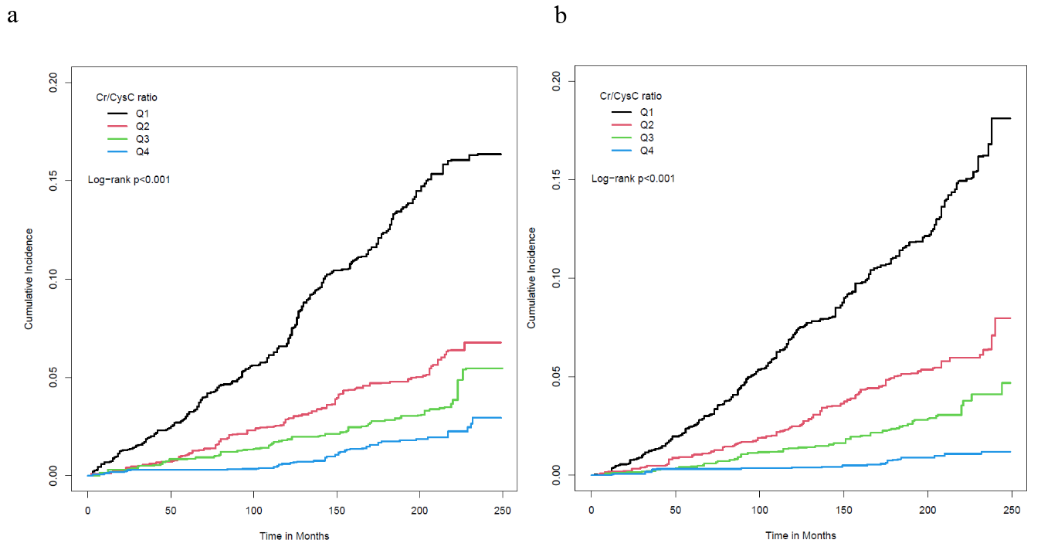


**Supplementary Figure 1. Cumulative incidence of CVD mortality presented by gender.** (a) Cumulative incidence of CVD mortality in male participates. (b) Cumulative incidence of CVD mortality in female participates.

**Supplementary table 1. Univariate and multivariate logistic regression model of Cr/CysC ratio (per SD increase) for CVD morbidity presented by gender**

| Outcomes | Unadjusted model | |  | Adjusted model^a^ | |
| --- | --- | --- | --- | --- | --- |
|  | OR^b^ (95% CI) | p-value |  | OR^b^ (95% CI) | p-value |
| **Male** |  |  |  |  |  |
| CVDs | 0.41 (0.35, 0.48) | <0.001 |  | 0.73 (0.55, 0.97) | 0.033 |
| Heart Failure | 0.36 (0.28, 0.46) | <0.001 |  | 0.42 (0.27, 0.65) | <0.001 |
| Coronary Heart Disease | 0.48 (0.41, 0.57) | <0.001 |  | 0.97 (0.79, 1.19) | 0.763 |
| Angina Pectoris | 0.42 (0.33, 0.54) | <0.001 |  | 0.75 (0.51, 1.09) | 0.122 |
| Myocardial Infarction | 0.44 (0.35, 0.56) | <0.001 |  | 0.73 (0.50, 1.07) | 0.100 |
| Stroke | 0.37 (0.29, 0.48) | <0.001 |  | 0.52 (0.35, 0.77) | 0.002 |
| **Female** |  |  |  |  |  |
| CVDs | 0.45 (0.39, 0.52) | <0.001 |  | 0.62 (0.49, 0.78) | <0.001 |
| Heart Failure | 0.31 (0.25, 0.38) | <0.001 |  | 0.39 (0.25, 0.61) | <0.001 |
| Coronary Heart Disease | 0.44 (0.33, 0.58) | <0.001 |  | 0.70 (0.44, 1.10) | 0.120 |
| Angina Pectoris | 0.52 (0.42, 0.65) | <0.001 |  | 0.83 (0.61, 1.11) | 0.198 |
| Myocardial Infarction | 0.44 (0.35, 0.56) | <0.001 |  | 0.69 (0.48, 1.00) | 0.053 |
| Stroke | 0.49 (0.36, 0.65) | <0.001 |  | 0.51 (0.35, 0.75) | 0.001 |

^a^ Adjusted for age, gender, and ethnicity, SBP, LDL-C, BMI, HbA1c, smoking, eGFR.

^b^ Odds ratio of Cr/CysC ratio (per SD increase)

Due to missing data, 768 participants are excluded from the adjusted model


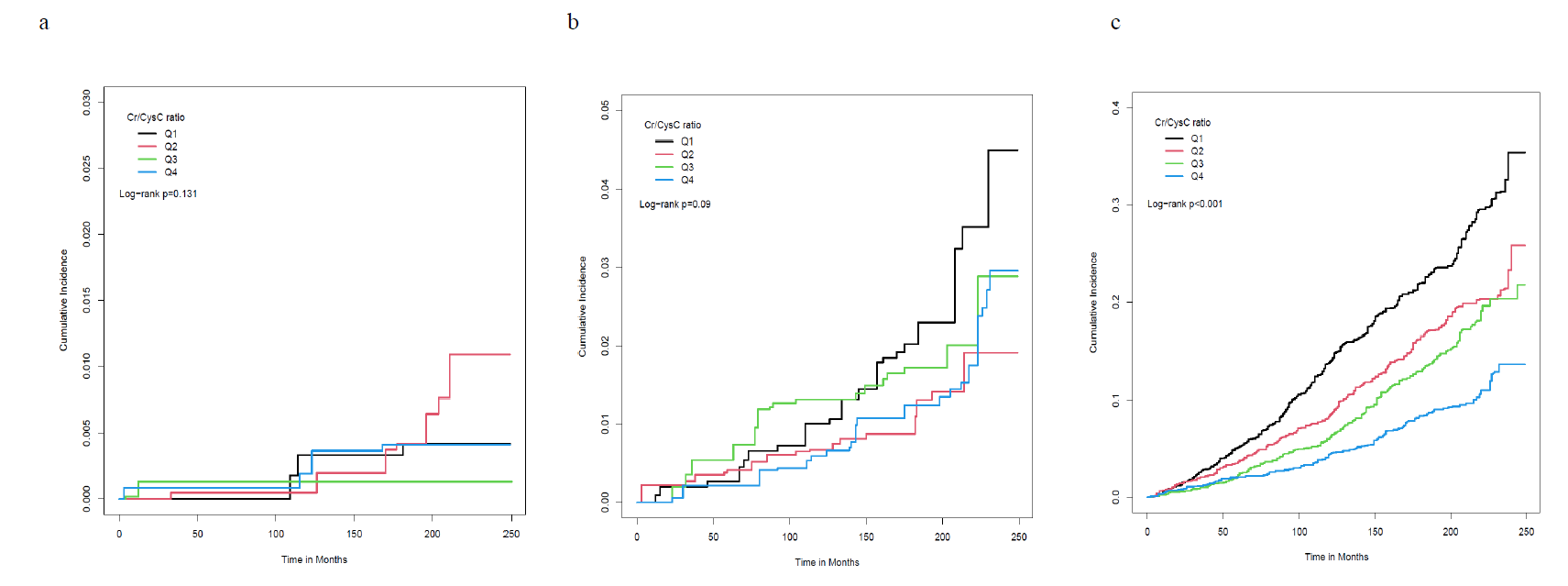


**Supplementary Figure 2. Cumulative incidence of CVD mortality presented by age.** (a) Cumulative incidence of CVD mortality in participates younger than 37 years older. (b) Cumulative incidence of CVD mortality in participates between 37 and 52 years older. (c) Cumulative incidence of CVD mortality in participates elder than 52 years older.

**Supplementary Table 2. Univariate and multivariate logistic regression model of Cr/CysC ratio (per SD increase) for CVD morbidity presented by age**

| Outcomes | Unadjusted model | |  | Adjusted model^a^ | |
| --- | --- | --- | --- | --- | --- |
|  | OR^b^ (95% CI) | p-value |  | OR^b^ (95% CI) | p-value |
| **Age tertile 1** |  |  |  |  |  |
| CVDs | 0.60 (0.44, 0.84) | 0.003 |  | 0.50 (0.28, 0.90) | 0.022 |
| Heart Failure | 0.60 (0.32, 1.12) | 0.108 |  | 1.01 (0.27, 3.72) | 0.993 |
| Coronary Heart Disease | 0.38 (0.26, 0.54) | <0.001 |  | 1.17 (0.90, 1.54) | 0.234 |
| Angina Pectoris | 0.91 (0.48, 1.73) | 0.763 |  | 0.64 (0.27, 1.53) | 0.308 |
| Myocardial Infarction | 0.66 (0.42, 1.04) | 0.071 |  | 0.80 (0.32, 2.01) | 0.631 |
| Stroke | 0.57 (0.37, 0.89) | 0.014 |  | 0.43 (0.18, 1.03) | 0.058 |
| **Age tertile 2** |  |  |  |  |  |
| CVDs | 0.57 (0.45, 0.73) | <0.001 |  | 0.61 (0.43, 0.86) | 0.007 |
| Heart Failure | 0.47 (0.28, 0.77) | 0.004 |  | 0.36 (0.16, 0.83) | 0.018 |
| Coronary Heart Disease | 0.86 (0.56, 1.31) | 0.472 |  | 0.96 (0.62, 1.48) | 0.839 |
| Angina Pectoris | 0.53 (0.37, 0.74) | <0.001 |  | 0.58 (0.32, 1.06) | 0.073 |
| Myocardial Infarction | 0.66 (0.45, 0.97) | 0.034 |  | 0.70 (0.39, 1.26) | 0.221 |
| Stroke | 0.50 (0.26, 0.93) | 0.030 |  | 0.50 (0.23, 1.09) | 0.079 |
| **Age tertile 3** |  |  |  |  |  |
| CVDs | 0.80 (0.70, 0.91) | <0.001 |  | 0.67 (0.53, 0.84) | 0.001 |
| Heart Failure | 0.56 (0.45, 0.68) | <0.001 |  | 0.39 (0.29, 0.54) | <0.001 |
| Coronary Heart Disease | 1.00 (0.89, 1.12) | 0.952 |  | 0.83 (0.63, 1.08) | 0.153 |
| Angina Pectoris | 0.87 (0.74, 1.02) | 0.089 |  | 0.77 (0.60, 0.98) | 0.034 |
| Myocardial Infarction | 0.95 (0.82, 1.12) | 0.550 |  | 0.69 (0.50, 0.97) | 0.032 |
| Stroke | 0.62 (0.50, 0.77) | <0.001 |  | 0.52 (0.38, 0.71) | <0.001 |

^a^ Adjusted for age, gender, and ethnicity, SBP, LDL-C, BMI, HbA1c, smoking, eGFR.

^b^ Odds ratio of Cr/CysC ratio (per SD increase)

Due to missing data, 768 participants are excluded from the adjusted model

Age tertile 1: participates younger than 37 years older; Age tertile 2: participates between 37 and 52 years older; Age tertile 3: participates elder than 52 years older

**Supplementary Table 3. Univariate and multivariate logistics regression model of Cr/CysC ratio (per SD increase) for CVD morbidity**

| Outcomes | Unadjusted model | |  | Adjusted model^a^ | |
| --- | --- | --- | --- | --- | --- |
|  | OR^b^ (95% CI) | p-value |  | OR^b^ (95% CI) | p-value |
| **CRP>=1.0 mg/dL** |  |  |  |  |  |
| CVDs | 0.48 (0.35, 0.65) | <0.001 |  | 0.45 (0.26, 0.79) | 0.007 |
| Heart Failure | 0.35 (0.23, 0.52) | <0.001 |  | 0.31 (0.16, 0.59) | <0.001 |
| Coronary Heart Disease | 0.42 (0.28, 0.63) | <0.001 |  | 0.39 (0.18, 0.86) | 0.021 |
| Angina Pectoris | 0.49 (0.30, 0.78) | 0.004 |  | 0.63 (0.32, 1.23) | 0.166 |
| Myocardial Infarction | 0.75 (0.49, 1.17) | 0.201 |  | 0.41 (0.19, 0.90) | 0.028 |
| Stroke | 0.30 (0.19, 0.47) | <0.001 |  | 0.19 (0.09, 0.37) | <0.001 |
| **CRP<1.0 mg/dL** |  |  |  |  |  |
| CVDs | 0.52 (0.46, 0.59) | <0.001 |  | 0.70 (0.55, 0.88) | 0.003 |
| Heart Failure | 0.43 (0.35, 0.53) | <0.001 |  | 0.42 (0.29, 0.61) | <0.001 |
| Coronary Heart Disease | 0.67 (0.57, 0.77) | <0.001 |  | 0.96 (0.80, 1.16) | 0.687 |
| Angina Pectoris | 0.56 (0.48, 0.67) | <0.001 |  | 0.79 (0.6, 1.05) | 0.097 |
| Myocardial Infarction | 0.59 (0.49, 0.71) | <0.001 |  | 0.76 (0.55, 1.05) | 0.094 |
| Stroke | 0.48 (0.38, 0.60) | <0.001 |  | 0.57 (0.40, 0.81) | 0.003 |

a Adjusted for age, gender, and ethnicity, SBP, LDL-C, BMI, HbA1c, smoking, eGFR.

b Odds ratio of Cr/CysC ratio (per SD increase)

**Supplementary Table 4. Univariate and multivariate logistics regression model of Cr/CysC ratio (per SD increase) for CVD morbidity**

| Outcomes | Unadjusted model | |  | Adjusted model^a^ | |
| --- | --- | --- | --- | --- | --- |
|  | OR^b^ (95% CI) | p-value |  | OR^b^ (95% CI) | p-value |
| **Malignancy** |  |  |  |  |  |
| CVDs | 0.52 (0.39, 0.69) | <0.001 |  | 0.44 (0.27, 0.74) | 0.003 |
| Heart Failure | 0.43 (0.24, 0.77) | 0.006 |  | 0.23 (0.09, 0.61) | 0.004 |
| Coronary Heart Disease | 0.72 (0.49, 1.07) | 0.098 |  | 0.71 (0.41, 1.26) | 0.233 |
| Angina Pectoris | 0.53 (0.35, 0.82) | 0.005 |  | 0.42 (0.22, 0.80) | 0.009 |
| Myocardial Infarction | 0.74 (0.52, 1.04) | 0.084 |  | 0.67 (0.39, 1.17) | 0.154 |
| Stroke | 0.45 (0.27, 0.75) | 0.003 |  | 0.43 (0.19, 0.97) | 0.042 |
| **Without malignancy** |  |  |  |  |  |
| CVDs | 0.53 (0.47, 0.59) | <0.001 |  | 0.68 (0.55, 0.85) | 0.001 |
| Heart Failure | 0.42 (0.35, 0.50) | <0.001 |  | 0.42 (0.32, 0.55) | <0.001 |
| Coronary Heart Disease | 0.65 (0.56, 0.74) | <0.001 |  | 0.91 (0.72, 1.14) | 0.398 |
| Angina Pectoris | 0.58 (0.49, 0.68) | <0.001 |  | 0.84 (0.65, 1.08) | 0.161 |
| Myocardial Infarction | 0.61 (0.51, 0.73) | <0.001 |  | 0.71 (0.51, 0.97) | 0.032 |
| Stroke | 0.46 (0.36, 0.57) | <0.001 |  | 0.50 (0.36, 0.69) | <0.001 |

^a^ Adjusted for age, gender, and ethnicity, SBP, LDL-C, BMI, HbA1c, smoking, eGFR.

^b^ Odds ratio of Cr/CysC ratio (per SD increase)

**Supplementary Table 5. Univariate and multivariate Cox regression analysis of Cr/CysC ratio (per SD increase) grouped by variables associated with CVD mortality.**

| Variables | Events/N at risk |  | Univariate analysis | |  | Multivariate analysis* | |
| --- | --- | --- | --- | --- | --- | --- | --- |
|  |  |  | HR (95% CI) | p-value |  | HR (95% CI) | p-value |
| CRP>=1.0 mg/dL | 370/1028 |  | 0.35 (0.22, 0.56) | <0.001 |  | 0.57 (0.32, 1.01) | 0.056 |
| CRP<1.0 mg/dL | 2449/9354 |  | 0.43 (0.38, 0.48) | <0.001 |  | 0.55 (0.46, 0.65) | <0.001 |
| Malignancy | 511/894 |  | 0.65 (0.45, 0.94) | 0.022 |  | 0.84 (0.50, 1.40) | 0.496 |
| Without malignancy | 2306/9481 |  | 0.40 (0.36, 0.45) | <0.001 |  | 0.50 (0.41, 0.60) | <0.001 |

*Adjusted for sex, age, ethnicity, SBP, LDL-C, BMI, HbA1c, smoking, eGFR, having heart failure, angina pectoris, myocardial infarction, coronary heart disease, stroke.
